# A Large Change in Temperature between Neighbouring Days Increases the Risk of Mortality

**DOI:** 10.1371/journal.pone.0016511

**Published:** 2011-02-02

**Authors:** Yuming Guo, Adrian G. Barnett, Weiwei Yu, Xiaochuan Pan, Xiaofang Ye, Cunrui Huang, Shilu Tong

**Affiliations:** 1 School of Public Health and Institute of Health and Biomedical Innovation, Queensland University of Technology, Brisbane, Australia; 2 Department of Occupational and Environmental Health, Peking University School of Public Health, Beijing, China; Centre de Recherche Public de la Santé (CRP-Santé), Luxembourg

## Abstract

**Background:**

Previous studies have found high temperatures increase the risk of mortality in summer. However, little is known about whether a sharp decrease or increase in temperature between neighbouring days has any effect on mortality.

**Method:**

Poisson regression models were used to estimate the association between temperature change and mortality in summer in Brisbane, Australia during 1996–2004 and Los Angeles, United States during 1987–2000. The temperature change was calculated as the current day's mean temperature minus the previous day's mean.

**Results:**

In Brisbane, a drop of more than 3°C in temperature between days was associated with relative risks (RRs) of 1.157 (95% confidence interval (CI): 1.024, 1.307) for total non-external mortality (NEM), 1.186 (95%CI: 1.002, 1.405) for NEM in females, and 1.442 (95%CI: 1.099, 1.892) for people aged 65–74 years. An increase of more than 3°C was associated with RRs of 1.353 (95%CI: 1.033, 1.772) for cardiovascular mortality and 1.667 (95%CI: 1.146, 2.425) for people aged <65 years. In Los Angeles, only a drop of more than 3°C was significantly associated with RRs of 1.133 (95%CI: 1.053, 1.219) for total NEM, 1.252 (95%CI: 1.131, 1.386) for cardiovascular mortality, and 1.254 (95%CI: 1.135, 1.385) for people aged ≥75 years. In both cities, there were joint effects of temperature change and mean temperature on NEM.

**Conclusion:**

A significant change in temperature of more than 3°C, whether positive or negative, has an adverse impact on mortality even after controlling for the current temperature.

## Introduction

Climate change is unequivocal, with a general increase in both mean temperature and temperature variability over the last half a century. These changes are primarily due to emissions of greenhouse gases caused by human activity [1], [2]. The frequency, intensity and duration of weather extremes (e.g. heat waves, floods and cyclones) are projected to increase as climate change continues [3], and unstable weather patterns (e.g. a significant drop/increase in temperature) are also more likely to occur in the coming decades [4]. As well as being an enormous environmental issue, climate change affects human health via extreme weather events and associated socio-ecological changes [2], [5].

Much recent research has assessed the relationship between temperature and human health. Morbidity and mortality are known to be seasonal, with excess morbidity and mortality during cold winters and hot summers [6], [7]. The effects of temperature on mortality and morbidity have been examined in various climates, and J-, V-, or U-shaped associations have been observed [8], [9], [10], [11], [12], [13]. However, less evidence is available on the possible effects on mortality due to temperature change between neighbouring days.

In this study we hypothesized that if the temperature changed sharply between neighbouring days, it would result in adverse impacts on human health. Because the effects of temperature are strongly dependent on season, we only analysed the relationship between temperature change and mortality in summer. Poisson regression models were used to examine the effects on mortality due to short-term changes in temperature between neighbouring days in Brisbane, Australia and Los Angeles, United States.

## Materials and Methods

### Data collection

Brisbane is the capital city of the state of Queensland in Australia, and is on the east coast of the country (27° 30′ south, 153° 00′ east). It has a humid subtropical climate, with the average temperature of 25°C in summer (Dec–Feb). Los Angeles is the largest city in the state of California and the Western United States (34° 03′ north, 118° 15′ west). Los Angeles has a dry-summer subtropical climate, with an average temperature of 21°C in summer (Jun–Aug). We chose these two cities, because they have a sub-tropical climate pattern and we aimed to explore whether the temperature change between neighbouring days has health effects in both the Northern and Southern Hemisphere.

We gathered the Brisbane data on daily deaths of non-external causes in summers between Jan, 1996 and Dec, 2004 from the Office of Economic and Statistical Research of the Queensland Treasury. The causes of non-external mortality (NEM) were coded according to the International Classification of Diseases, ninth version (ICD-9) (ICD-9: 001–799) before December 1996 and tenth version (ICD-10) (ICD-10: A00–R99) between December 1996 and December 2004. Cardiovascular mortality (CVM; ICD-9:390–459, ICD-10:I00–I79) and respiratory mortality (RM; ICD-9: 460–519, ICD-10:J00–J99) were extracted from the mortality database. Influenza deaths (ICD-9: 487.0–487.8 or ICD-10: J10–J11) were excluded from respiratory mortality. All deaths were for residents of Brisbane city. We stratified NEM by gender and age (3 groups: 0–64, 65–74, and ≥75 years).

We gathered daily meteorological data including mean temperature and mean relative humidity (RH) from the Australian Bureau of Meteorology. Values of temperature change were calculated using the current day's mean temperature minus the previous day's mean temperature. Temperature change between the neighbouring days is a measure of temperature stability, with large positive and negative values indicating an unstable temperature. The air pollutants including daily mean ozone (O_3_) and particulate matter less than 10 µm in aerodynamic diameter (PM_10_) were monitored at a central site in Brisbane. We collected these data from the Queensland Environmental Protection Agency.

Los Angeles' data were obtained from the National Morbidity and Mortality Air Pollution Study (NMMAPS) which is publicly available and covers the years 1987 to 2000. Mean temperature, relative humidity, O_3_, NEM, CVM, RM, and NEM in age groups (0–64, 65–74, and ≥75 years) were used here. We excluded PM_10_, because there was a large number of missing values. Mortality counts were not split by gender in the NMMAPS, so the impact of temperature change on mortality by gender could not be analysed in Los Angeles.

### Data analysis

Poisson generalized additive models (GAMs) were used to examine the effects of short-term changes in temperature between neighbouring days on mortality. We used GAMs because the associations between temperature change and mortality are non-linear, and the daily number of deaths has an over-dispersed Poisson distribution. We adjusted for day of the week (DOW) using a categorical variable. Regression splines for calendar time and year were used to control for long-term trends and seasonal patterns [14]. We controlled for relative humidity, PM_10_, and O_3_ using regression splines.

To assess the effects of temperature change on mortality, we used the following model:
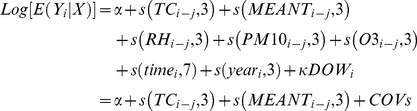
(1)where *i* is the day of the observation; *j* is the lag days; *E(Y_i_|X)* are the estimates of daily death counts on day *i*; α is the intercept; *s* (, γ) is a regression spline with γ degrees of freedom. *TC_i-j_* is temperature change, *MEANT_i-j_* is daily mean temperature, *RH_i-j_* is relative humidity, *PM_10i-j_*, is particulate matter, and *O_3i-j_* is ozone; *time_i_* is days of calendar time on day *i*; *year_i_* is the year on day *i*; *DOW_i_* is the day of the week on day *i*, and **κ** is vector of coefficients for *DOW*; *COVs* represents all other covariates in the model.

As an alternative model to compare the effects of large changes in temperature on mortality with moderate changes, temperature change was categorised into 3 groups: a drop of more than 3°C; a rise of more than 3°C; a change in either direction of less than 3°C. Model (1) was altered by modifying the single terms of *TC_i-j_* into a categorical variable as follows:
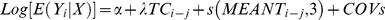
(2)where *TC_i-j_* is a categorical variable, **λ** is vector of coefficients for categories of temperature change.

The joint effects of temperature change and mean temperature were estimated using GAMs. We plotted these estimates to assess whether there was an interaction between temperature change and mean temperature on mortality. Model (1) was modified by changing the single terms of *TC_i-j_* and *MEANT_i-j_* into a bivariate term as follows:
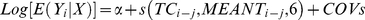
(3)where *s* (*TC_i-j_*, *MEANT_i-j_*,6) is the joint effect of temperature change and mean temperature on mortality, which we modelled using a regression spline with 6 degrees of freedom.

The Akaike information criterion (AIC) was used to measure goodness of fit. Residuals were examined to evaluate the adequacy of the models. Sensitivity analyses were performed through changing degrees of freedom for time and removing PM_10_ from the Brisbane data. Relative risks (RRs) and confidence intervals (CIs) were calculated. All statistical tests were two-sided. Values of *P*<0.05 were considered statistically significant. Spearman correlation coefficients were used to summarize the correlations between daily weather conditions and air pollutants in each city. The R software (version 2.10.1, R Development Core Team 2009) was used to fit all models.

## Results

Table 1 summarises the daily weather conditions, air pollutants, and mortality in summers in Brisbane from 1996 to 2004 and Los Angeles from 1987 to 2000. The temperature change ranged from −6.5°C to 5.0°C in Brisbane, and from −5.3°C to 5.8°C in Los Angeles. The mean temperature was higher in Brisbane (24.4°C) than in Los Angeles (21.3°C). There were, on average, 16 daily deaths from non-external causes in Brisbane, and 138 in Los Angeles.

**Table 1:** Summary statistics for daily weather conditions, air pollutants, and mortality in Brisbane, Australia and Los Angeles, United States.

City	Variable	Frequency distribution					Mean	SD	Sum
		Min	25%	Median	75%	Max			
Brisbane	TC (°C)	−6.5	−0.6	0.1	0.8	5.0	0.01	1.2	—
	MEANT (°C)	18.8	23.2	24.4	25.6	31.9	24.4	1.8	—
	RH (%)	38.9	68.0	73.4	79.1	97.5	73.5	8.3	—
	O_3_ (ppb)	0.0	8.0	10.8	14.0	45.	11.4	5.2	—
	PM_10_ (µg/m^3^)	3.9	12.6	15.5	19.1	84.5	16.9	7.4	—
	NEM	1	13	15	18	43	16	4.4	12,364
	CVM	0	5	6	8	31	6	2.9	5,076
	RM	0	0	1	2	6	1	1.1	916
	Age <65 years	0	2	3	4	12	3	1.7	2,372
	Age 65–74 years	0	1	2	4	11	3	1.8	2,133
	Age ≥75 years	1	8	10	10	12	37	3.5	7,859
	Male	1	5	8	9	20	8	2.9	6,093
	Female	1	6	8	9	30	8	3.0	6,271
Los Angeles	TC (°C)	−5.3	−0.56	0	0.56	5.8	0	1.0	—
	MEANT (°C)	14.3	20.0	21.1	22.4	29.4	21.3	2.1	—
	RH (%)	34.2	71.9	76.3	79.6	89.6	75.2	6.8	—
	O_3_ (ppb)	−18.2	4.9	10.0	15.2	44.9	10.2	7.9	—
	NEM	95	12.9	138	147	217	138	13.4	177,384
	CVM	34	57	63	70	106	64	9.4	81,913
	RM	3	9	11	14	22	12	3.6	14,917
	Age <65 years	17	34	39	44	70	39	7.4	50,163
	Age 65–74 years	14	25	28	32	50	29	5.8	37,021
	Age ≥75 years	44	64	70	76	109	70	9.4	90,200

Table 2 shows the Spearman's correlations between daily weather conditions and air pollutants. Temperature change was positively correlated with mean temperature in both cities. In Brisbane, there were no statistically significant correlations between temperature change and humidity, while temperature change was negatively correlated with humidity but positively with O_3_.

**Table 2:** Spearman's correlation between daily weather conditions and air pollutants in Brisbane, Australia and Los Angeles, United States.

	Brisbane				Los Angeles		
	MEANT	TC	RH	O_3_	MEANT	TC	RH
TC	0.30**				0.20**		
RH	0.21**	0.05			−0.10**	−0.28**	
O_3_	0.19**	0.03	−0.15**		0.04	0.17**	0.21**
PM_10_	0.20**	0.07	−0.24**	0.40**	——	——	——

**P<0.01.

**Figure 1:**
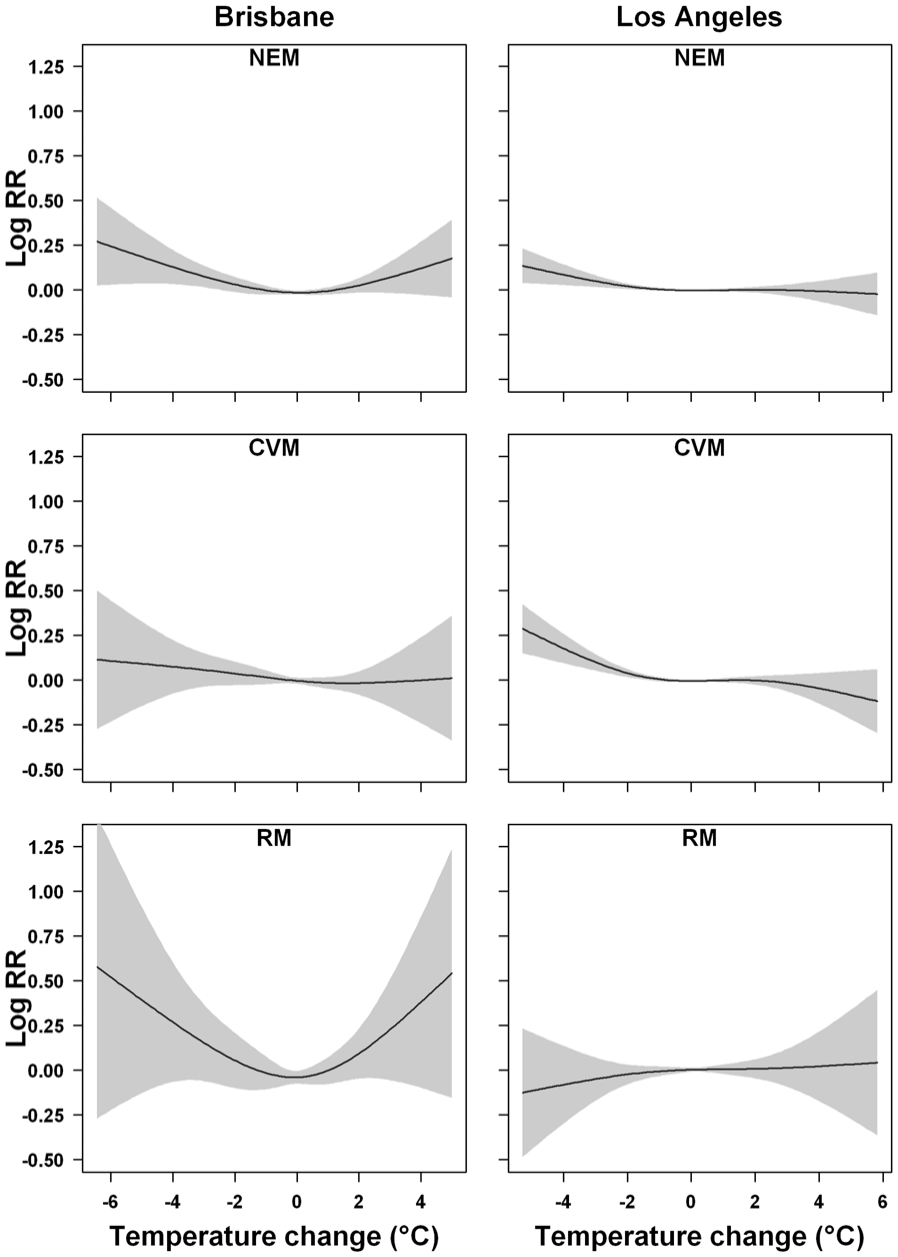
The associations between temperature change and non-external mortality, cardiovascular mortality, and respiratory mortality using model (1) in Brisbane, Australia (left side) and Los Angeles, United States (right side).

In both cities, there was little effect of temperature change on mortality, when the temperature change ranged from −3°C to 3°C (Figure 1). Therefore, we divided temperature change into three categories: less than −3°C, −3°C to 3°C, and more than 3°C.

**Figure 2:**
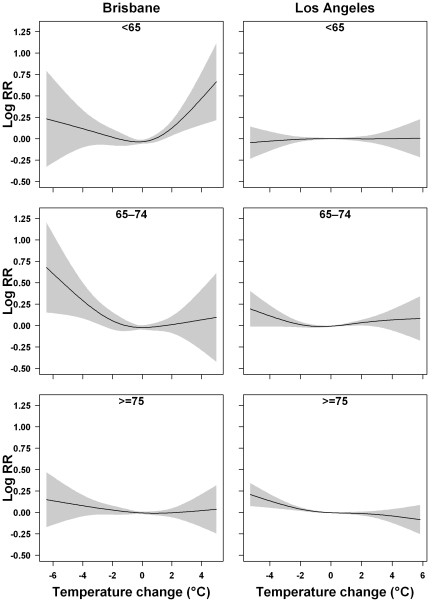
The associations between temperature change and non-external mortality by age group using model (1) in Brisbane, Australia (left side) and Los Angeles (right side), United States.

Figure 2 shows the association between temperature change and NEM by age group. In Brisbane, people aged <65 years were vulnerable to a sharp increase in temperature, while those aged 65–74 years were sensitive to a sudden drop in temperature. In Los Angeles, both people aged 65–74 and ≥75 years were vulnerable to a sudden temperature drop, while no significant effects of temperature change were found for those aged <65 years.

The delayed effect of temperature change on mortality was examined using model (1). The change in temperature between current day and previous day (lag 0) had the highest impact on current day's mortality (results not shown). Therefore, we only show the associations between temperature change and mortality on the current day (Table 3). As a continuous variable, temperature change only had statistically significant effects on CVM and NEM in those aged ≥75 years in Los Angeles but not on other groups. However, further analyses using model (2) show that a temperature drop of more than 3°C had statistically significant adverse impacts on total NEM, NEM among those aged 65–74 years, and women in Brisbane; on total NEM, CVM, and NEM among those aged ≥75 years in Los Angeles. A temperature increased of more than 3°C was significantly associated with CVM and NEM among those aged <65 years in Brisbane.

**Table 3:** The associations between temperature change and mortality in Brisbane, Australia and Los Angeles, United States.

		RR (95% CI)		
		1°C increase in TC (°C)^a^	TC <−3°C^b^	TC >3°C^b^
Brisbane	NEM	0.993 (0.977, 1.008)	1.157 (1.024, 1.307)**	1.198 (0.997, 1.438)
	CVM	0.986 (0.962, 1.011)	1.115 (0.923, 1.347)	1.353 (1.033, 1.772)**
	RM	0.997 (0.941, 1.057)	1.202 (0.774, 1.867)	1.608 (0.925, 2.794)
	Age<65 years	1.021 (0.985, 1.059)	1.135 (0.859, 1.501)	1.667 (1.146, 2.425)**
	Age 65–74 years	0.971 (0.935, 1.009)	1.442 (1.099, 1.892)**	1.016 (0.631, 1.634)
	Age ≥75 years	0.990 (0.971, 1.010)	1.088 (0.930, 1.273)	1.118 (0.885, 1.413)
	Male	1.001 (0.978, 1.024)	1.131 (0.949, 1.348)	1.225 (0.941, 1.596)
	Female	0.985 (0.963, 1.007)	1.186 (1.002, 1.405)*	1.174 (0.910, 1.513)
Los Angeles	NEM	0.994 (0.989, 1.000)	1.133 (1.053, 1.219)**	1.039 (0.971, 1.112)
	CVM	0.988 (0.979, 0.997)**	1.252 (1.131, 1.386)**	1.031 (0.933, 1.140)
	RM	1.008 (0.988, 1.029)	1.006 (0.767, 1.321)	1.002 (0.792, 1.266)
	Age<65 years	1.001 (0.990, 1.013)	0.957 (0.825, 1.108)	1.014 (0.893, 1.153)
	Age 65–74 years	1.004 (0.991, 1.018)	1.092 (0.929, 1.284)	1.106 (0.955, 1.280)
	Age ≥75 years	0.986 (0.978, 0.995)**	1.254 (1.135, 1.385)**	1.026 (0.933, 1.129)

*P<0.05;

**P<0.01;

a TC as a continuous variable, using model (1);

b TC as a categorical variable, using model (2).

**Figure 3:**
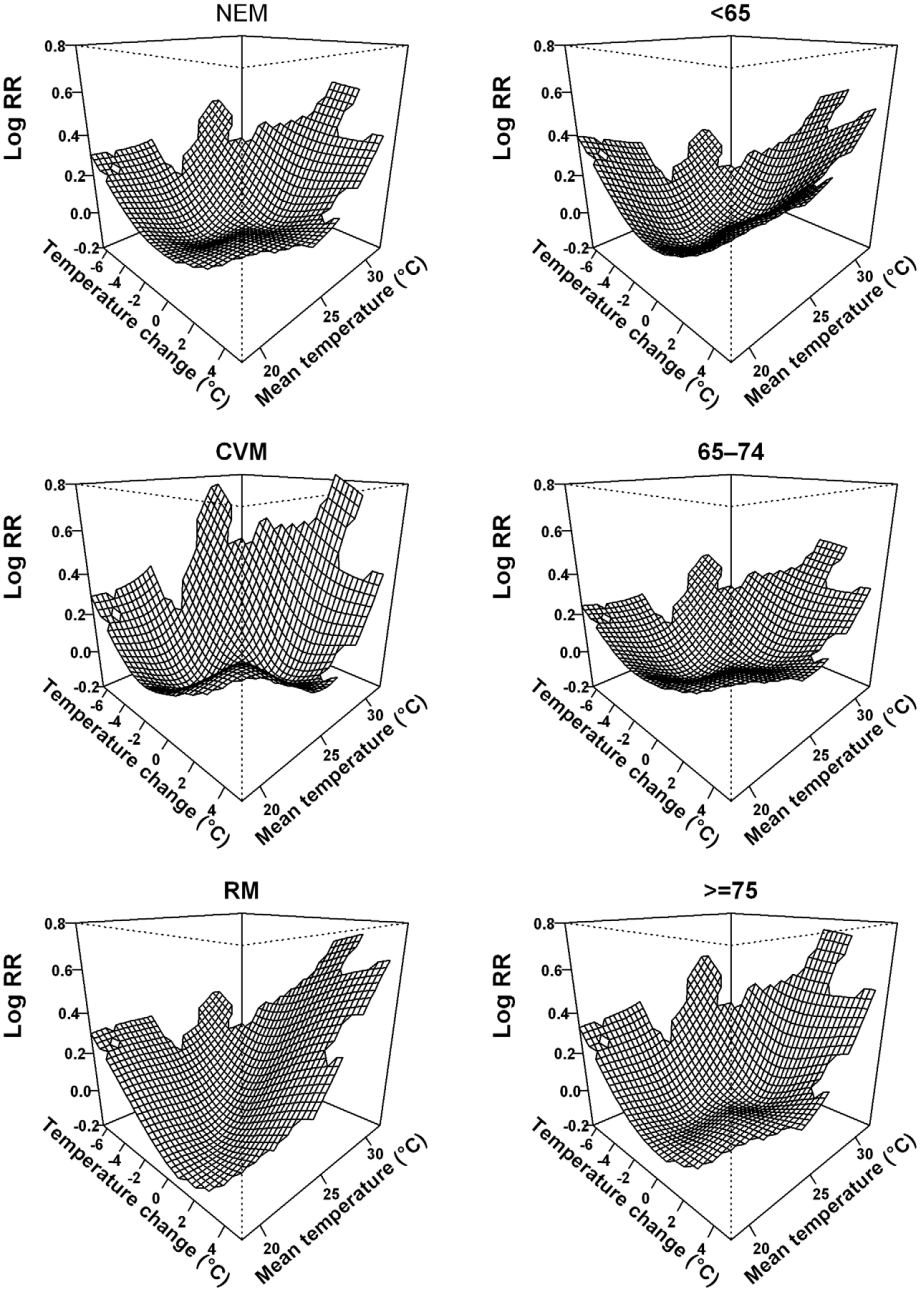
Bivariate response surfaces of the temperature change and mean temperature for non-external mortality, subgroups of mortality using model (3) in Brisbane, Australia.

**Figure 4:**
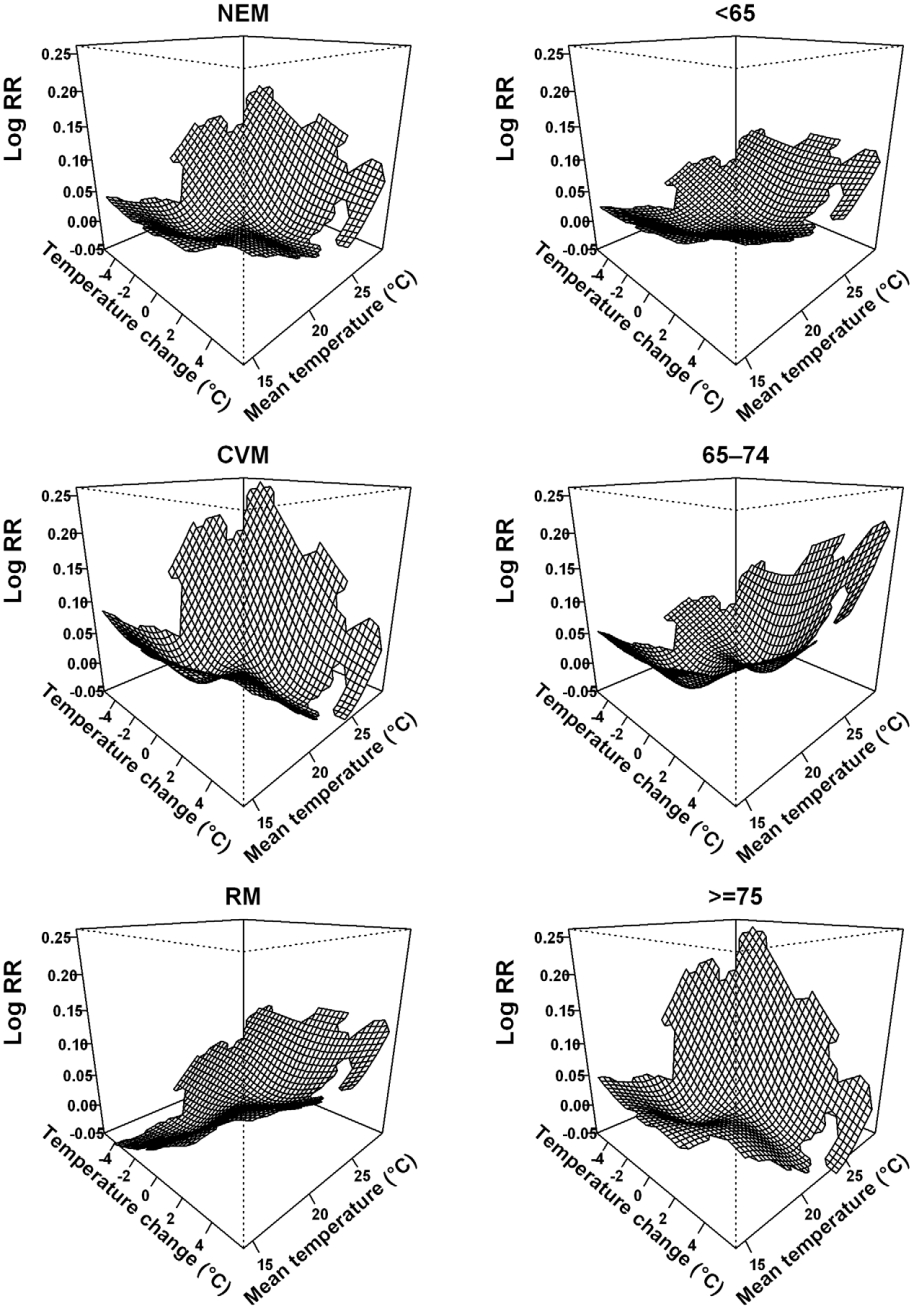
Bivariate response surfaces of the temperature change and mean temperature for non-external mortality, subgroups of mortality using model (3) in Los Angeles, United States.

Figure 3 and Figure 4 illustrate the joint effects of temperature change and mean temperature on NEM and subgroups of NEM using model (3). The adverse effects of mean temperature on mortality occurred when the mean temperature was under 26°C in Brisbane and under 24°C in Los Angeles. In contrast, when we used model (1), mean temperature had no adverse effect on mortality in the temperature range under 26°C in Brisbane and 24°C in Los Angeles (Figure S1 and Figure S2). The J-shaped relationships between mean temperature and mortality in model (1) become approximately U-shaped relationships when the joint effect of temperature change and mean temperature was modelled (except for RM and NEM among those aged ≥75 years in Brisbane). These results suggest that there were joint effects of temperature change and mean temperature on mortality.

In order to perform sensitivity analyses, we changed degrees of freedom for time and removed PM_10_ from Brisbane data. The results showed that there were no substantial change in effect estimates. Also, the residual analyses showed that models were a good fit to the data.

## Discussion

This study examined the effect of temperature change on mortality, and explored the joint effects of temperature change and mean temperature on mortality in Brisbane, Australia, and Los Angeles, United States. In Brisbane, a relatively large decrease in temperature between neighbouring days increased the risk of total NEM, and NEM among those aged 65–74 years and in women overall. A sharp increase in temperature was significantly associated with increased CVM and NEM among those aged <65 years. A significant drop in temperature increased the risks of total NEM, CVM, and NEM among those aged ≥75 in Los Angeles. Also, joint effects of temperature change and mean temperature on mortality were found in both locations.

These increased risks of death during periods of temperature fluctuations highlight the importance of not only considering hot absolute temperatures in relation to human health, but also sudden changes in temperature, particularly for a relatively large temperature changes (more than 3°C).

We assessed whether temperature change had an adverse impact on mortality in different subtropical climates and in different locations. Both Brisbane and Los Angeles have a subtropical summer climate, but Brisbane is humid while Los Angeles is dry. The non-linear pattern in the increased risk of mortality for a change in temperature was similar in the two cities, although there were some differences. These differences might be caused by population characteristics (e.g. racial composition), geographic location, and living conditions including air conditioning and family income, as well as access to health care [15].

Some previous studies have examined the effects of sudden changes in temperature on cardiovascular disease, and found similar results. For example, Kyobutungi et al. [16] investigated the relationship between ischemic stroke occurrence and the temperature change in 24 hours, but without controlling for season. The results showed that sudden temperature changes of more than 5°C, regardless of whether the change was negative or positive, were associated with an increased risk of acute ischemic stroke. Schneider et al. [17] carried out a longitudinal study to examine the impact of weather parameters on cardiovascular patients. Results showed that a rise or fall in air temperature was associated with an increase in heart rate. Ebi et al. [18] found that a 3°C increase in minimum temperature or decrease in maximum temperature caused a significant increase in hospital admissions for cardiovascular diseases and stroke in three Californian regions, with a stronger association for the oldest age group. However, Plavcova et al. [19] only found a significant increase in mortality for large increases in temperature.

A large change in temperature might impact on mortality, whether it is positive or negative, because the automatic thermoregulation system cannot adapt to sudden temperature change, particularly for people with certain medical conditions. Sudden changes in temperature have been associated with risk factors for human health, such as increases in blood cholesterol levels, blood pressure, plasma fibrinogen concentrations, peripheral vasoconstriction, heart rate, platelet viscosity, and reducing the immune system's resistance [17], [20], [21].

In this study, we found females were more sensitive to a drop in temperature than males in Brisbane. Previous studies have found that gender can modify the association between temperature and health [22], [23], [24], [25]. There is evidence that women are more vulnerable to heat-related mortality than men [22], [23], [24], [25]. Studies have also found that women have higher risks for ischemic, arrhythmic and blood pressure effects associated with the weather [26], [27]. However, Basu [28] pointed out that the differences of the effect of temperature on women and men was dependent on location and population. For example, the impact of hot temperature on mortality was higher for men in São Paulo, but higher for women in Mexico City [29].

The association between temperature change and mortality varied by age group, and the effect of age differed between Brisbane and Los Angeles. This may be due to different life styles, living conditions, family income, as well as access to health care. Many studies have shown that age is a modifier of the association between temperature and health [22], [24], [25]. Keatinge et al. [30] found that people aged 65–74 years were the most vulnerable subgroup to cold in seven European countries. Hajat et al. [31] found that the elderly were the most vulnerable group to temperature in England and Wales, both for cold and hot weather.

Our findings suggest that people with cardiovascular diseases are more vulnerable to short-term changes in temperature than those with respiratory diseases (figure 3 and figure 4). Many studies have shown that temperature is associated with physiological changes in the circulatory system, including blood pressure, heart rate, blood cholesterol levels, plasma fibrinogen concentrations, peripheral vasoconstriction, and platelet viscosity [17], [20], [21]. These factors are directly associated with cardiovascular function. Respiratory mortality is generally attributed to the immune system's resistance to respiratory infections caused by exposure to cold or hot temperatures [8]. Therefore, people with some pre-existing cardiovascular disease might be more sensitive than those with pre-existing respiratory disease to short-term changes in temperature.

We controlled for mean temperature as many studies have illustrated a consistent relationship between temperature and human health [8], [9], [10], [11], [12], [13]. Saez et al. found a 1°C increase in temperature was associated with 1.7%, 4.2%, and 13.2% increase in NEM, CVM, and RM respectively [32]. Schwartz found that people with cardiovascular diseases, chronic obstructive pulmonary disease, or diabetes appeared more vulnerable to the effects of hot weather [33]. Mean temperature was also associated with mortality in the present study (Table S1). The mechanisms of heat-related deaths may result from failure in the thermoregulation which may be impaired by dehydration, salt depletion and increased surface blood circulation during hot period [28], [34]. Elevated blood viscosity, cholesterol levels and sweating thresholds might also trigger heat-related mortality [28], [35]. The reduced sweat gland output and skin blood flow, reduction in cardiac output and less redistribution of blood flow from renal and splanchnic circulations will impair thermoregulation. We only examined the effect of mean temperature, not maximum, minimum, and apparent temperature. A recent study has shown how these different measures of temperature gave similar results for predicting mortality, so we would anticipate similar results if we used alternative temperature measures [36].

This study has four strengths. To our knowledge, this is the first study to examine whether temperature change has an adverse effect on mortality in summer. We also explored whether there were joint effects of temperature change and mean temperature on mortality. We examined the impacts of temperature change on NEM in several subcategories. We used two cities in different countries with different subtropical climates to confirm the findings.

This study also has some limitations. The findings of this study may not be generalisable to other locations, particularly places with different climates. We used the data on temperature and air pollution from fixed sites rather than individual exposure. Therefore, there might be the potential for exposure measurement bias.

In conclusion, we found there were adverse effects due to relatively large changes in temperature on NEM, particularly for females and people aged 65–74 years in summer in Brisbane, as well as on NEM, CVM, and NEM among people aged ≥ 75 years in Los Angeles. A significant increase in temperature was also associated with CVM and NEM in those <65 years in Brisbane. In addition, there were joint effects of temperature change and mean temperature on NEM and most subgroups in both cities. These findings suggest that people should not only pay attention to the increases in absolute temperature in summer, but also to temperature changes of 3°C of more. The findings might provide an important impetus for evaluating population vulnerability, and improving the climate change adaption strategies.

## Supporting Information

Figure S1 The associations between the mean temperature and non-external mortality, cardiovascular mortality, and respiratory mortality using model (1) in Brisbane, Australia (left side) and Los Angeles, United States (right side).
(TIF)

Figure S2 The associations between the mean temperature and age groups of non-external mortality using model (1) in Brisbane, Australia (left side) and Los Angeles, United States (right side).
(TIF)

Table S1 The associations between a 1°C increase in mean temperature and mortality in Brisbane, Australia and Los Angeles, United States.
(DOC)
