# Supplementary figures and images for: A Large Change in Temperature between Neighbouring Days Increases the Risk of Mortality
Source: PLoS One. 2011 Feb 2;6(2):e16511. doi: 10.1371/journal.pone.0016511 (PMC3032790; doi:10.1371/journal.pone.0016511)

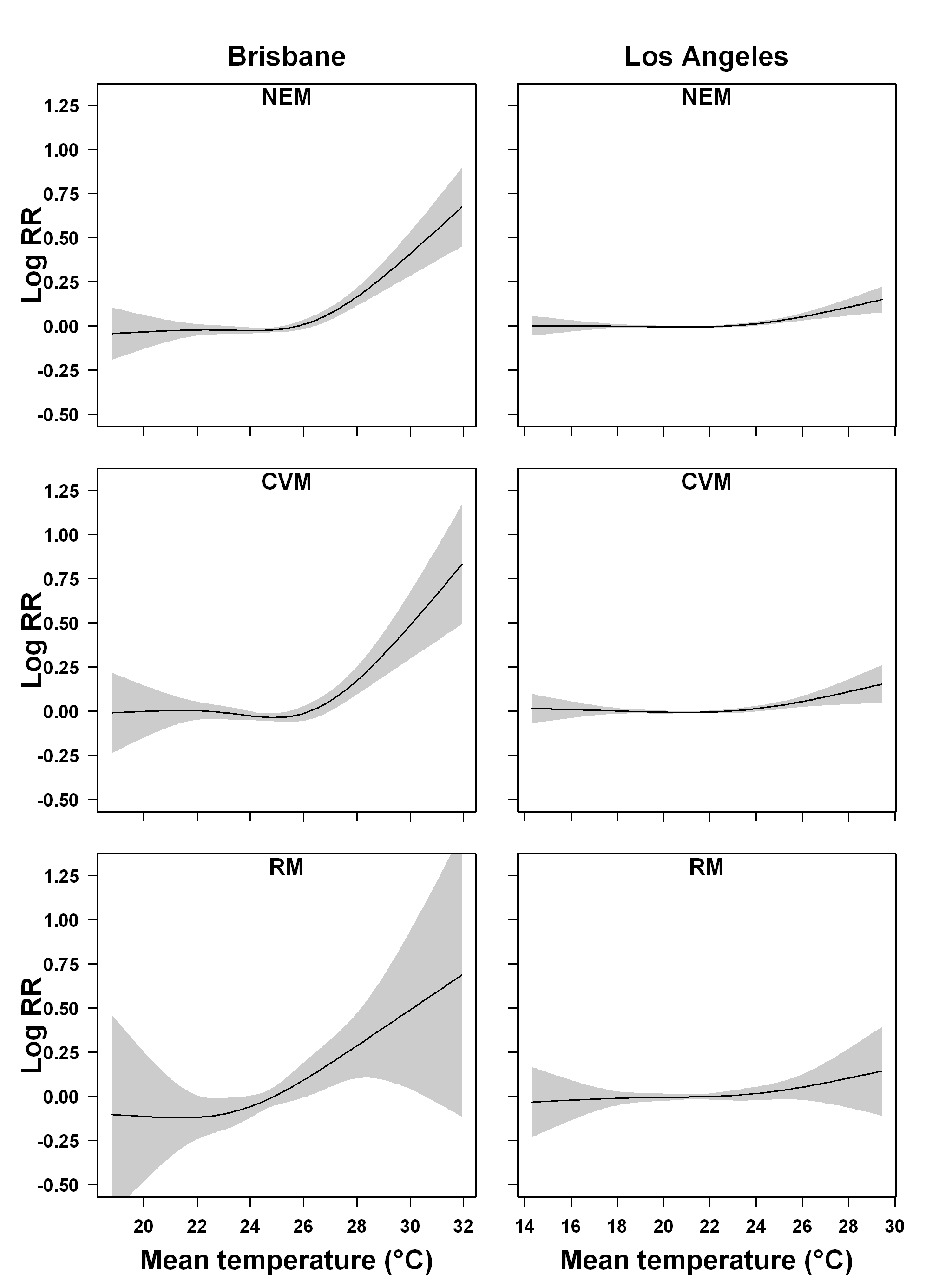

Supplement: Figure S1 — The associations between the mean temperature and non-external mortality, cardiovascular mortality, and respiratory mortality using model (1) in Brisbane, Australia (left side) and Los Angeles, United States (right side). (TIF) [file pone.0016511.s001.tif]

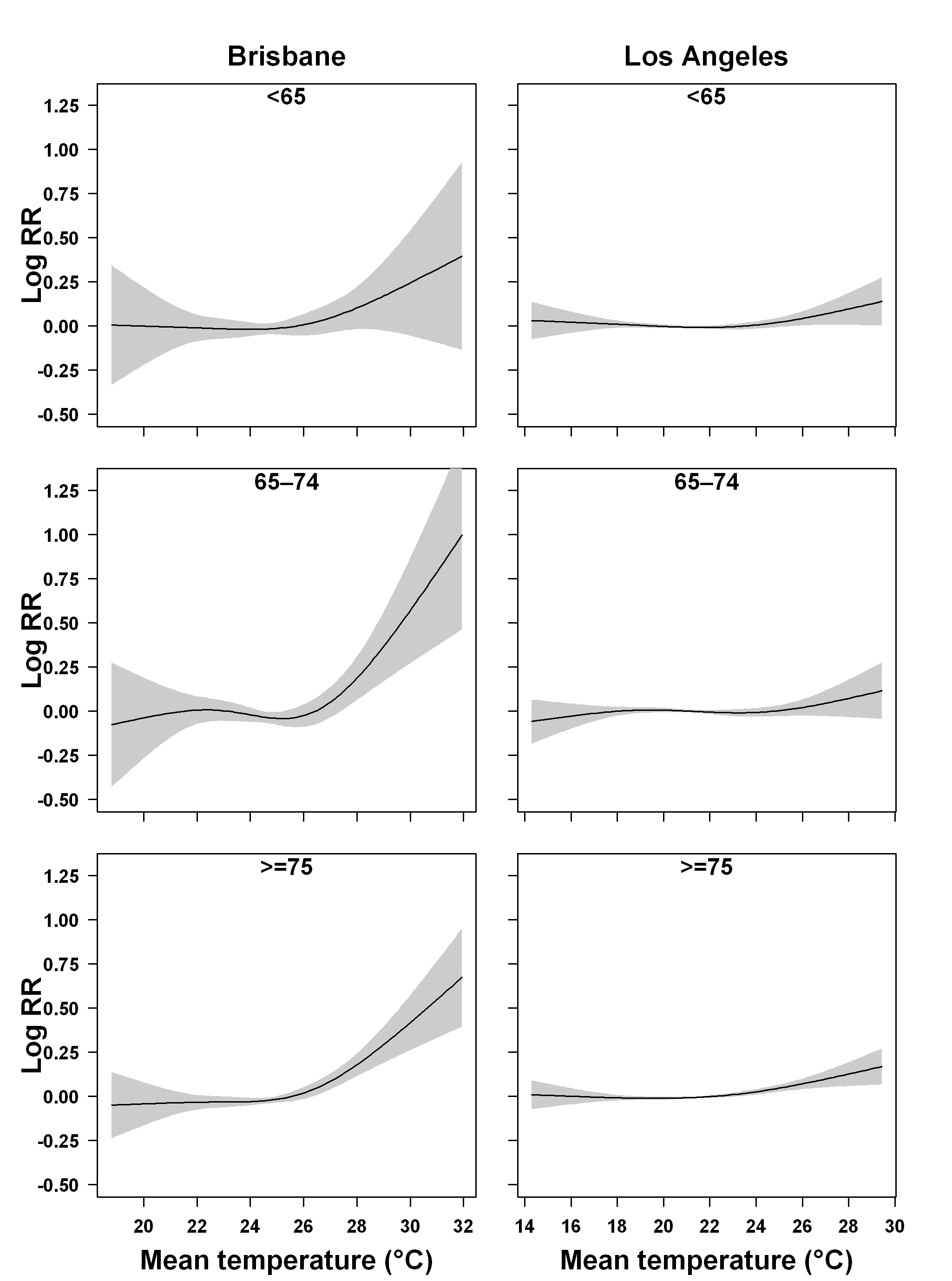

Supplement: Figure S2 — The associations between the mean temperature and age groups of non-external mortality using model (1) in Brisbane, Australia (left side) and Los Angeles, United States (right side). (TIF) [file pone.0016511.s002.tif]
