# Supplementary material for: A Large Change in Temperature between Neighbouring Days Increases the Risk of Mortality
Source: PLoS One. 2011 Feb 2;6(2):e16511. doi: 10.1371/journal.pone.0016511 (PMC3032790; doi:10.1371/journal.pone.0016511)
Supplement: Table S1 — The associations between a 1°C increase in mean temperature and mortality in Brisbane, Australia and Los Angeles, United States. (DOC) [file pone.0016511.s003.doc]

Table S1: The associations between a 1 °C increase in mean temperature and mortality in Brisbane, Australia and Los Angeles, United States

|  | RR (95% CI) | | |
| --- | --- | --- | --- |
|  | Brisbane |  | Los Angeles |
| NEM | 1.029 (1.017, 1.041)** |  | 1.004 (1.001, 1.007 )** |
| CVM | 1.026 (1.008, 1.044)** |  | 1.004 (1.000, 1.008) |
| RM | 1.066 (1.023, 1.110)** |  | 1.006 (0.996, 1.016) |
| Age <65 years | 1.019 (0.993, 1.045) |  | 1.001 (0.996, 1.007) |
| Age 65–74 years | 1.031 (1.003, 1.059)* |  | 1.001 (0.995, 1.007) |
| Age ≥75 years | 1.034 (1.019, 1.049)** |  | 1.008 (1.003,1.012)** |
| Male | 1.020 (1.004, 1.036)* |  | ——————— |
| Female | 1.036 (1.019, 1.053)** |  | ——————— |

***P*<0.01; **P*<0.05
